# Bioelectric profiling of *Rickettsia montanensis* in Vero cells utilizing dielectrophoresis

**DOI:** 10.1186/s13036-025-00487-y

**Published:** 2025-02-18

**Authors:** Negar Farhang Doost, Sai Deepika Reddy Yaram, Kayla Wagner, Harshit Garg, Soumya K. Srivastava

**Affiliations:** 1 Department of Chemical and Biomedical Engineering, West Virginia University, 1306 Evansdale Dr., PO Box 6102, Morgantown, WV 26506-6102 USA; 2 Department of Biochemical Engineering and Biotechnology, Indian Institute of Technology- Delhi, Delhi, India

**Keywords:** Dielectrophoresis, Electrophysiological properties, Bioelectric signatures, Diagnostic tool, Rocky mountain spotted fever, *Rickettsia montanensis*

## Abstract

Rickettsia is an intracellular bacteria transmitted to humans through ticks, lice, fleas, or their feces, causing acute symptoms such as fever, headache, rashes, and muscle aches. Detecting rickettsial diseases is challenging due to limitations in current methods such as negative results, low sensitivity, and high cost. These limitations highlight the need for improved detection methods. Dielectrophoresis (DEP) offers a promising alternative to develop a point-of-care economical, label-free, and sensitive diagnostic tool. By exposing cells to non-uniform electric fields one can measure the electrical properties of the cells which are different and unique based on the cell type. By comparing the dielectric profiles of healthy and infected cells, DEP could be utilized to design a rapid, cost-effective diagnostic tool. Initial steps involve characterizing the electrophysiological properties of Vero cells infected with *Rickettsia montanensis* to develop this new detection tool. This study found significant differences in electrical parameters between healthy and *Rickettsia spp.* infected Vero cells, particularly at a medium conductivity of 500 µS/cm. Moreover, we found that the dielectric spectrum showed the greatest differences between healthy and *Rickettsia spp.* infected Vero cells at medium conductivity of 500 µS/cm, with significantly different dielectrophoretic crossover frequencies (no DEP force region). These findings suggest that dielectrophoretic detection of infected cells could serve as a quick, cost-effective, label-free, and sensitive alternative for developing a point-of-care diagnostic tool for Rickettsial infections.

**Supplementary Information:**

The online version contains supplementary material available at 10.1186/s13036-025-00487-y.

## Introduction

Rickettsia are a group of intracellular bacteria found in ticks, lice, fleas, etc., transmitted by a bite or the feces of these infected organisms, which cause acute nonspecific symptoms like fever, headache, rashes, muscle aches, etc. in humans [1]. Over 5,000 cases of Rocky Mountain Spotted fever (RMSF) were reported in the United States in 2019, which is a minor drop from the peak of over 6,000 cases in 2017. Nonetheless, from 2000 to 2017, the prevalence of all rickettsial illnesses increased steadily. The chances of developing RMSF are highest in those over 40 years of age, while the risk of death is highest in children under 10 years of age. People 60 and older accounted for the largest number of Spotted Fever Rickettsioses (SFR) cases in 2019. The mortality rate for RMSF has decreased dramatically over the past century due to the development of tetracycline antibiotics; however, it can still reach to 30% in untreated cases [2–4]. The detection of rickettsial diseases varies and depends on the type and timing of specimen collection. Commonly, detection is through molecular methods like polymerase chain reaction (PCR) tests or using serological techniques like indirect immunofluorescence antibody (IFA) assay [1]. These methods have various shortcomings, which makes rickettsial diseases challenging to detect and, thus, the neglected nature of these infections [5]. The serological methods detect the antibodies in the pathogen but require convalescent and acute samples to confirm the disease. Thus, within the first week after the symptoms develop, when the patients need the most medical care, these serological techniques will likely yield negative results. After the first week of symptoms, most patients start recovering and no longer require medical care and thus would not go for a diagnosis. On the other hand, molecular techniques detect the direct presence of pathogens. Therefore, they can detect the disease quickly, but the cost and maintenance required for the laboratory setup are high and make it impossible for endemic use scenarios. In most cases, antibiotic therapy is started before the diagnostic tests, which decreases the sensitivity and makes detection difficult. These limitations of the current detection methods emphasize the need for better detection techniques as the diagnosis in the acute phase is rare, affecting the treatment of an increasing disease (1.7 patients per million person-years (PY) in 2000 to 14.3 patients per million PY in 2012) [5, 6].

However, these limitations can be addressed by using a microfluidic electrokinetic technique, dielectrophoresis (DEP). Dielectrophoresis provides significant benefits for the controlled manipulation, enrichment, separation, and analysis of biological samples, offering a noninvasive, nondestructive, and rapid approach [7]. Dielectrophoresis is observed when cells are exposed to non-uniform electric fields across a wide frequency range, allowing for the determination of electrical properties such as membrane capacitance, conductance, and cytoplasm conductivity. DEP has been used to qualitatively quantify the bioelectrical signatures of excitable cells and benchmarks well against other validated methods [8]. DEP-spectra has also successfully defined biomarkers for stem cell differentiation [9], cancer [10, 11], infections of red blood cells [12], red blood cell storage [13], and rare earth elements bioaccumulation [14, 15]. However, although DEP is most effective for measuring cell electrical properties, its adoption as a method in general cell biology has been limited by electrode technology and the challenges associated with robust data analysis. Additionally, impedance flow cytometry and dielectrophoresis (DEP) are both powerful electrokinetic techniques used to analyze and manipulate cells based on their electrical properties. Impedance flow cytometry enables the label-free characterization of cells by measuring changes in electrical impedance as they pass through a microfluidic channel with embedded electrodes. This technique provides real-time assessment of cell size, membrane integrity, and intracellular conductivity in a continuous flow environment.

In contrast, DEP leverages the dielectric properties of cells to induce their movement within a non-uniform electric field, allowing for selective manipulation and separation based on differences in polarizability. This method is particularly effective in distinguishing between infected and healthy Vero cells, as it exploits variations in membrane capacitance and cytoplasmic conductivity. Such differences arise due to pathogen-induced alterations in cellular structure and ion exchange dynamics. As a result, DEP serves as a highly sensitive tool for investigating rickettsial infections and other intracellular pathogens by providing label-free, non-invasive discrimination of infected cells [16, 17]. In this research, we utilize a 3DEP analyzer to overcome some of these challenges to characterize the cells under a single frequency at a time to generate a complete dielectric spectrum. Studying the differences in dielectric profiles between healthy and infected cells enables the development of a rapid and cost-effective diagnostic tool.

Analyzing the dielectric profiles of healthy and infected cells using dielectrophoresis enables the assessment of changes in electrophysiological properties, such as membrane conductance and capacitance, and their correlation with alterations in cell morphology and cytoplasmic composition. These changes are crucial as they can provide insights into disease mechanisms and potentially lead to the development of rapid and cost-effective diagnostic tools. Characterizing the electrophysiological properties of infected and uninfected cells is a necessary first step to understanding how these alterations correlate with disease progression [18]. In this research, we utilize Vero cells as hosts for *Rickettsia montanensis* and obtain dielectric profiles or bioelectric signatures before and after infection. Vero cells are particularly susceptible to many germs due to their interferon expression deficiency, which impairs their anti-viral defense mechanism, making the Vero cells an appropriate host [19]. Other continuous cell lines (CCLs) include MDCK, HEK-293, PER.C6, CAP, AGE1.CR, and EB66 are also utilized by other researchers, but the extensive experience with Vero cells and their acceptance by regulatory authorities make them ideal candidates for many vaccine manufacturers [20].

## Theory

Dielectrophoresis is the process of subjecting dielectric particles to a non-uniform alternating (AC) or direct current (DC) electric field. Direct current dielectrophoresis (DC DEP or iDEP) uses a stationary electric field and insulating objstaples to create a non-uniformity in the electric field [21–23]. Alternating current dielectrophoresis (AC DEP) utilizes an alternating electric field to generate a non-uniform field, which will be employed in this study [24, 25]. As the AC frequency is applied at a fixed peak-to-peak voltage, the behavior of the cells suspended in the medium changes. This physical phenomenon is due to the transient electric dipoles that arise in areas of electric field gradient as a result of the applied electric field [8]. A cell’s behavior under this non-uniform electric field are used to extract the dielectric properties such as membrane capacitance and conductance, as well as cytoplasm conductivity and permittivity.

The net dielectric force a spherical particle experiences can be calculated using [13]:

1 $${F_{DEP}} = \:2\pi \:{r^3}{\varepsilon _{med}}Re[K(\omega \:)]\nabla \:{E^2}$$

where *r* is the cell radius, $\:\epsilon\:$_med_ is the suspending medium permittivity, $\:{\epsilon\:}_{o}$is the permittivity of the free space, $\:\nabla\:{E}^{2}$ is the gradient of the electric field, and $\:Re\left[K\right(\omega\:\left)\right]$ is the real part of the Clausius-Mossotti factor discussed below.

The Clausius-Mossotti (CM) factor is a mathematical relationship that relates the dielectric properties of the surrounding medium to that of the cell. This factor defines the cell motion due to the force acting on cells under the non-uniform electric field [26]. The CM factor can be calculated by [13]:

2 $$\:K\left( \omega \right)\: = \:\frac{{\varepsilon _{cell}^* - \varepsilon _{med}^*}}{{\varepsilon _{cell}^* + 2\varepsilon _{med}^*}}$$

where *med* and *cell* refer to the suspending medium and particle respectively, and $\:\epsilon\:$^*^ refers to the complex permittivity given by [13]:

3 $${\varepsilon ^*}\: = \:\varepsilon \: - \:j\frac{\sigma }{\omega }$$

where $\:\omega\:$ is the angular frequency of the applied field, $\:\epsilon\:$ is the permittivity, $\:\sigma\:$ is the conductivity, and $\:j$ is the complex number $\:\sqrt{-1}$.

**Fig. 1:**
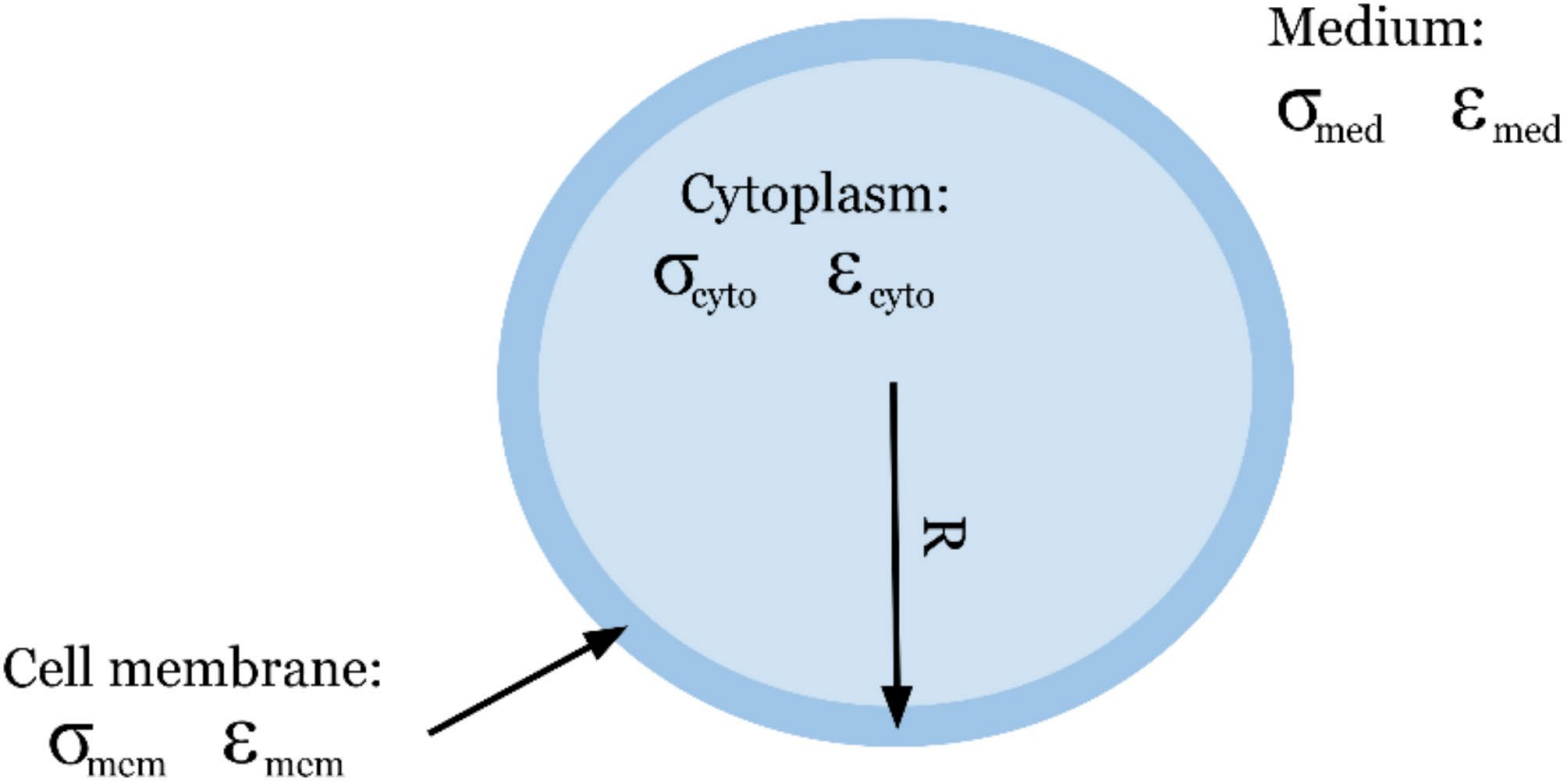
Dielectric single-shell model depicting the cell membrane (mem), cytoplasm (cyto), and suspending medium (med). Each component has a corresponding conductivity and permittivity

To relate the dielectric properties of the cell, the structure and morphology of the cell must be understood to model the properties and tie them to the biology accurately. Since Vero cells are composed of a cell membrane that contains the cytoplasm as well as other organelles, a single-shell dielectric model was used in this study [10]. The cell membrane and cytoplasm of the cell have their own dielectric properties, i.e., conductivity and permittivity, as depicted in Fig. 1. Equation 4 is referred to as the single-shell model, which relates the properties of the membrane and cytoplasm to the suspending medium as described in Eq. 2 [18].

4 $$\:\varepsilon _{cell}^*\: = \varepsilon _{mem}^*\frac{{{{\left( {\frac{r}{{r - t}}} \right)}^3} + 2\frac{{\varepsilon _{cyto}^* - \varepsilon _{mem}^*}}{{\varepsilon _{cyto}^* + 2\varepsilon _{mem}^*}}}}{{{{\left( {\frac{r}{{r - t}}} \right)}^3} + \frac{{\varepsilon _{cyto}^* - \varepsilon _{mem}^*}}{{\varepsilon _{cyto}^* + 2\varepsilon _{mem}^*}}}}\:$$

where *r* is the radius of the cell, *t* is the thickness of the cell, and *mem* and *cyto* refer to membrane and cytoplasm respectively.

DEP force depends on the frequency of the applied electric field. Prior to the application of AC voltage, particles are seen to be floating freely in the medium, but as the AC signal is turned on, the cells experience negative dielectrophoresis (nDEP), positive dielectrophoresis (pDEP), or the crossover state. The cells move away from the electrode edge, thus experiencing nDEP and this occurs when the cell’s polarizability is less than that of the medium [26]. The cells are attracted toward the electrode edge (wall of the microwell in the 3DEP dielectrophoretic cytometer used in this study), experiencing pDEP, occurring when the cell’s polarizability is greater than that of the medium. The frequency at which the cells reverse the direction is the crossover state, at which the DEP force is zero.

A plot of DEP force vs. applied frequency can be used to analyze the dielectric behavior of cells at varying frequencies. When the DEP force is less than zero, the cells experience nDEP, and the DEP force is positive when cells are attracted toward a high electric field region. The crossover frequency occurs when the DEP force is zero as cells transition from nDEP to pDEP or vice versa. The first crossover frequency point is dependent on the cell morphology, i.e., size, shape, and outer membrane morphology. The medium conductivity affects the first crossover frequency which transitions from nDEP to pDEP. The second crossover frequency occurs when cells transition from pDEP to nDEP and is dependent on the cell’s interior physiology with minimal dependency on the conductivity of the suspending medium [26]. When the applied electric field is adjusted to match the crossover frequency, it is possible to manipulate these particles by controlling their behavior, i.e., whether they are attracted or repelled from the electrodes, to separate, trap, or sort particles based on their dielectric properties. Dielectrophoresis (DEP) is an effective method for manipulating biological particles, offering label-free, cost-effective, and low-damage separation based on particle’s phenotype, genotype, and electrical properties. This approach provides precise and controlled handling of biological samples [27]. DEP spectral measurements are performed using the 3DEP dielectrophoretic cytometer in conjunction with a DEPwell 806 chip (DepTech, Uckfield, U.K.). The 3DEP system enables rapid and accurate cell analysis by qualitatively characterizing the cell movement yielding bioelectric signatures. It includes a chip with 20 microwells connected to a reader, with each microwell capable of generating electric fields at varying frequencies (up to 45 MHz). Cells suspended in the microwells redistribute according to their dielectric properties, with changes in light absorption measured to determine their electrical characteristics. This high-throughput system collects data within seconds, facilitating efficient, low-impact, and scalable analysis for applications such as studying cell behavior and the associated electrical properties. A detailed explanation of the equipment is provided elsewhere [8].

## Materials and methods

### Cell culture and sample preparation

Vero cells, the epithelial cells derived from normal adult African green monkeys, are the host cells for *Rickettsia montanensis* infection. The frozen stock of Vero cells was purchased from ATCC, USA, and stored in a -80 ^0^C freezer. Dulbecco’s Modified Eagle Medium (DMEM) supplemented with 10% heat-inactivated fetal bovine serum (FBS) was used for cell cultivation [28]. 10 mL of supplemented media was added to the 75 cm^2^ tissue culture flask, along with the thawed frozen sample of Vero cells. The flasks were incubated at 37 °C and 5% CO_2_ in a CO_2_ incubator (CB-S 170, Binder, Germany). Media renewal and passage occurred 2–3 times a week. Cells were monitored every day to reach a >90% confluent monolayer which usually takes 5–6 days.

Once the cells were confluent enough, the growth media was replaced with 9 mL of fresh media. A frozen sample of *R. montanensis* M5/6 received from Rocky Mountain Laboratory (NIH) was thawed and 200 µL was added to the flask. Infected cells were incubated at 34 ^0^C and 5% CO_2_. Cells were monitored every day until heavy infection level was achieved that often took 2–3 days. Microscopic examinations of Vero cells were performed every day, both prior to and following infection with *Rickettsia montanensis*. The cells maintained normal morphology and adherence, demonstrating sustained viability throughout the experimental period.

**Fig. 2:**
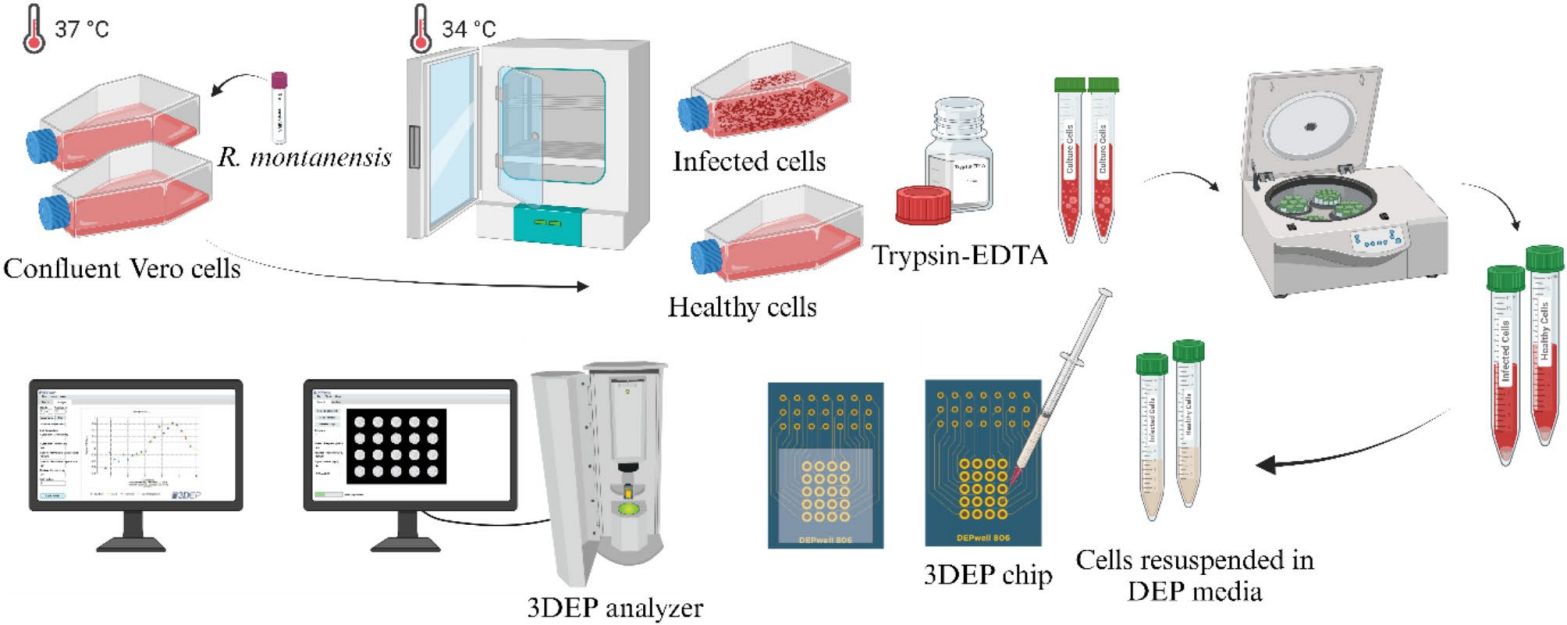
Sample preparation for analyzing dielectric behavior utilizing 3DEP Analyzer equipment. The culturing protocol was established according to ATCC. The suspending medium for dielectrophoretic experiments was adjusted for pH and conductivity, and osmolarity was maintained at the physiological conditions

Once the cells reached the confluent monolayer or heavy infection levels, the growth media was carefully removed. Then, the cells were detached from the culture vessel by adding 5 mL of 1× Trypsin-EDTA solution and incubated for 3–5 min at 37 °C to ensure complete detachment as shown in Fig. 2. Previous studies, such as Mahabadi et al. [29], have confirmed that Trypsin treatment is a safe and effective method, primarily causing cell rounding without significantly altering dielectric properties or inducing mechanical damage. In contrast, alternative methods like scraping can cause notable changes in cell morphology and dielectric properties, making Trypsin the preferred choice for preserving cell integrity.

The cell suspension was collected and centrifuged (Z206A, Hermle, USA) at 200 × g for 5 min. After the centrifugation, the supernatant was carefully removed, and the cell pellet was resuspended in the prepared DEP suspending media that is described below. This step was repeated three times to ensure there was no residue in the growth media. Finally, the cell pellet was resuspended in 5 mL of DEP suspending media.

### DEP experimentation

The DEP suspending media was prepared by dissolving 8.5 g of sucrose and 0.3 g of dextrose in 100 mL of deionized water (DI). Phosphate buffer saline (PBS) was utilized to adjust the conductivity of the media to approximately 100, 200, 300, and 500 µS/cm using a conductivity meter (InLab 731-ISM, Mettler Toledo, USA). This recipe contains high sugar to maintain the physiological osmolarity and PBS to stabilize the pH.

The cell suspension (~ 80 µL) obtained after washing and resuspension in DEP media was injected into the 3DEP chip (DEPwell 806, DEPtech, Uckfield, UK) with a 1 mL syringe and 25G needle (Air-Tite, USA) as shown in Fig. 2. The chip was covered with 25 × 25 mm glass coverslip to avoid the formation of meniscus. The chip was inserted into the 3DEP equipment (DEPtech, Uckfield, UK), as demonstrated in Fig. 2. To prevent exposure to ambient room light, the door was carefully checked to ensure it was fully latched. The light intensity on the 3DEP analyzer was adjusted to a setting of 24. The frequency range and voltage were set to 0.5–45000 kHz and 10 V_pp_, respectively. The applied peak-to-peak voltage of 10 V_pp_ corresponds to an electric field strength significantly below the threshold reported for electroporation in Vero cells, where membrane permeabilization typically occurs [30–32]. Joule heating can result in temperature increases that may affect cell viability and experimental outcomes. However, the voltage and frequency parameters used in this study fall within ranges demonstrated to produce minimal Joule heating effects. For instance, studies have shown that at similar voltage levels, the temperature rise is negligible, posing no risk to biological samples [33].

Research indicates that DEP force, when applied within specific parameters, do not adversely affect cell viability. For example, Hoettges et al. demonstrated that the 3DEP platform maintained cell health across various cell types, even under experimental conditions similar to those in this study [8]. Similarly, studies on apoptosis progression using DEP confirmed that cells remained viable during analysis [34]. In this study, we adhered to established protocols for voltage and frequency to minimize any impact on cell viability. Additionally, the short exposure duration (60 seconds) significantly reduced the likelihood of adverse effects.

In the 3DEP software, the analysis period was set to 30 s, medium permittivity to 78 F/m, and conductivity adjusted accordingly for each sample run to 100, 200, 300, or 500 µS/cm. The cell radius was defined as 3.32 μm for healthy cells and 5.55 μm for infected cells after imaging the cells by Transmission Electron Microscopy (TEM) as discussed in Sect. 3.4.

### Data collection and statistical analysis

Experimental data for healthy Vero cells was collected initially, followed by the *Rickettsia*-infected Vero cells at varying suspending medium conductivities and at a fixed peak-to-peak AC voltage. Data was gathered from 3 biological and 10 technical replicates using the 3DEP analyzer (DepTech, Uckfield, U.K.), with an R^2^-value (goodness-of-fit) exceeding 0.9. The data generated from the 3DEP analyzer is automatically fitted using a single-shell model by adjusting the model parameters to minimize the error between experimental data and theoretical predictions. The software utilizes optimization algorithms, such as least squares, to refine membrane permittivity, cytoplasm permittivity and conductivity, and the effective radius. The statistical analysis was performed using GraphPad Prism 10.2.3. Initially, experimental data for healthy and infected cells for each dielectric property were tabulated in GraphPad Prism and then a t-test for paired data that compares the means within the same group under two conditions, i.e., before and after infection, was performed to obtain statistical significance, i.e., by computing the p-value. Subsequently, the data were plotted with the standard error of the mean (SEM) displayed.

### Transmission electron microscopy (TEM)

Healthy and *Rickettsia montanensis*-infected Vero cells were cultured as mentioned in Sect. 3.1. and subsequently fixed with 3% glutaraldehyde for 12–24 h. Following fixation, samples were rinsed three times in 0.1 M cacodylate media for 10 min per rinse. Post-fixation was performed in a 1% osmium tetroxide and 0.8% potassium ferricyanide solution for 1.5 h. Samples were rinsed again three times in 0.1 M cacodylate media, 10 min per rinse. Dehydration was carried out in a graded ethanol series of 50%, 75%, and 95%, for 10 min each. Samples were further dehydrated in two changes of 100% ethanol for 10 min each. Infiltration began with a 2:1 acetone/resin mixture for 1 h, with gentle stirring on a rotator or stir plate. This was followed by infiltration with a 1:1 acetone/resin mixture for 1 h under the same conditions. A final infiltration was performed using a 1:2 acetone/resin mixture for 1 h, with continuous stirring. Samples were then infiltrated with two changes of pure resin under vacuum for 1 h each. The tissue was cross-sectionally embedded and cured at 70 ^0^C in an oven overnight. After sectioning, a strip of parafilm was placed on a hard, clean surface. Drops of uranyl acetate solution were dispensed onto the parafilm using a syringe fitted with a syringe filter, with one drop allocated per grid to be stained. The grids were placed into the uranyl acetate drops, ensuring the section side was facing either up or down. The grids were stained for 2 min. The grids were thoroughly washed in distilled water by placing drops of distilled water on the parafilm, with one drop per grid. The grids were submerged in the distilled water drops for 1 min, section side up or down. The washing steps were repeated twice for a total of three washes. The grids were placed on filter paper to dry. A new piece of parafilm was prepared. Drops of lead citrate solution were placed on the new parafilm using a syringe with an attached syringe filter, with one drop per grid to be stained. The grids were then placed in the lead citrate drops, with the section side facing either up or down. The grids were left under the lead citrate drops for 2 min. The grids were washed thoroughly in distilled water by placing drops of distilled water on the parafilm, with one drop per grid. The grids were submerged in the distilled water drops for 2 min, section side up or down. The washing steps were repeated twice for a total of three washes. The grids were placed on filter paper to dry. Finally, TEM was performed on the stained grids. Both sample preparation for TEM and SEM (scanning electron microscopy) imaging were conducted at the Health Sciences Facility of West Virginia University.

## Results and discussion

Vero cells obtained from the kidney of an African green monkey [28], are frequently employed as hosts for the replication of *Rickettsia spp*. It is important to detect rickettsial disease at the early stages as failure to administer timely treatment may lead to serious illness, hospitalization, and consequences such as hearing loss, enduring cognitive impairment, limb removal, and fatality [28]. Dielectrophoretic cell sorting represents an advanced technique employing electric fields to manipulate and segregate cells according to their electrical characteristics selectively, which can be used to develop a diagnostic tool to detect rickettsial diseases in a label-free manner [35]. The first step in designing a diagnostic platform is to analyze the cell behavior under a non-uniform electric field to obtain the dielectric properties of cells under varying suspending medium conductivity. The results of the analyzed data to obtain the bioelectric signatures of healthy and infected Vero cells using the dielectrophoretic crossover technique and dielectric single-shell model is discussed along with the TEM analysis to accurately determine the radius and thickness of both healthy and infected cells.

### Transmission electron microscopy (TEM) analysis

**Fig. 3:**
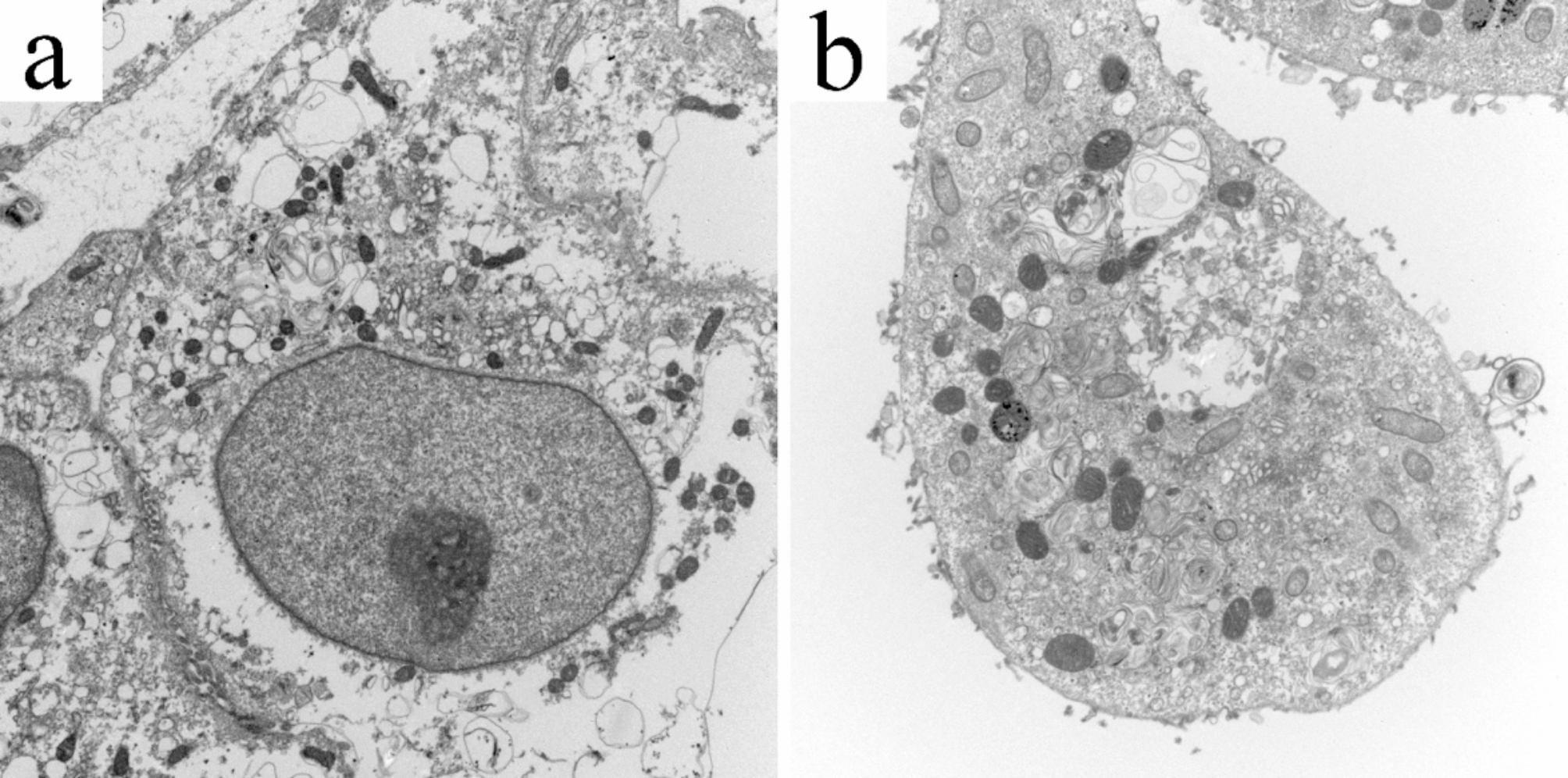
Transmission Electron Microscopy of healthy and infected Vero cells. **(a)** Non-infected (healthy) Vero cells ((magnification ×7500). **(b)***Rickettsia montanensis*-infected Vero cells (magnification ×10000). The diameters of healthy and infected Vero cells were 6.65 μm and 11.11 μm, respectively. The membrane thicknesses were measured to be 16 nm and 18 nm, for the healthy and infected cells, respectively.

TEM was performed at the Health Sciences Facility at West Virginia University, Morgantown, WV, to obtain the diameter and thickness of the healthy and infected rickettsial cells, and the images are shown in Fig. 3. The infection inside the cell can be seen in Fig. 3**(b)**. *Rickettsia spp.* are obligate intracellular parasitic bacteria that internalize within minutes. They escape from phagosomes into the cytoplasm through the phospholipase activities of hemolysin C (TlyC) and phospholipase D [36]. The diameter and membrane thickness are critical parameters that must be considered for the analysis of the single-shell model (refer to Eq. 4), as discussed earlier in the theory section (Sect. 2).

**Fig. 4:**
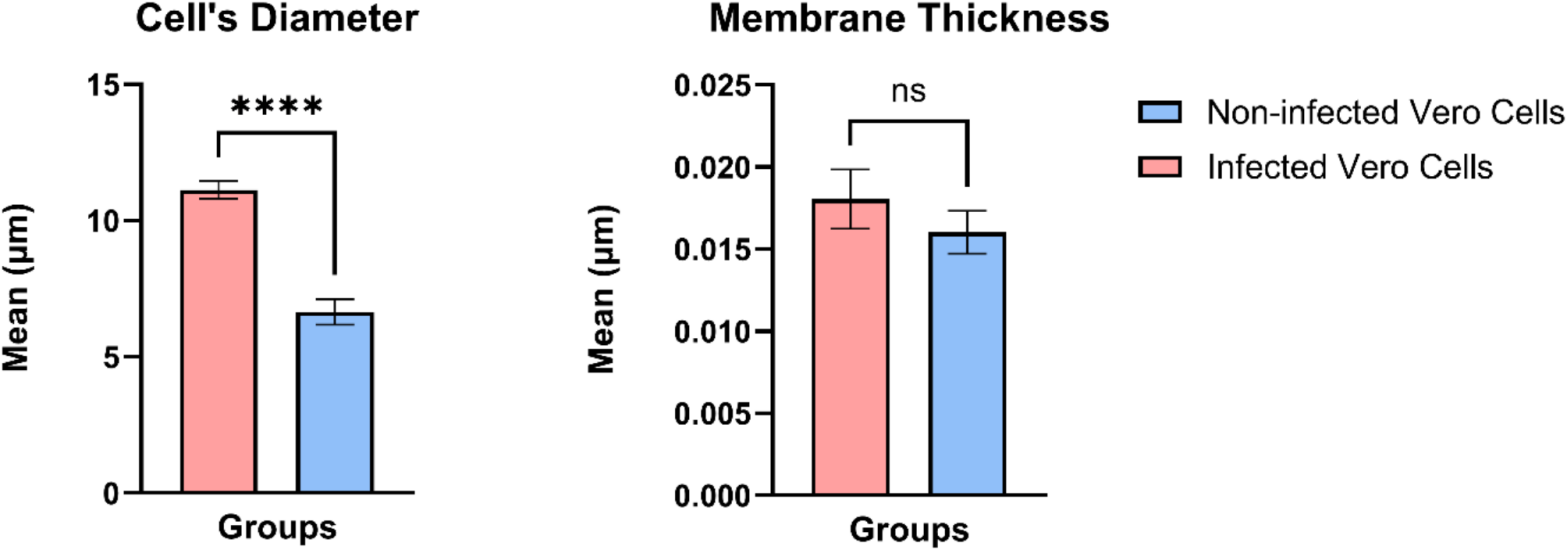
Cell diameter and membrane thickness for the healthy and infected Vero cells across 8 cell images obtained from Transmission Electron Microscopy. The cell diameter between the infected and healthy cells were statistically distinct whereas there were no significant differences observed in membrane thickness.

The images in Fig. 3 were utilized to obtain the diameters of healthy and infected Vero cells and were found to be 6.65 μm and 11.11 μm, respectively. The membrane thicknesses of these cells were measured to be 16 nm and 18 nm, for the healthy and infected cells, respectively, as determined by the TEM analysis. This significant increase in the diameter is likely due to the accumulation of bacteria within the cytoplasm, leading to cell swelling [37], as well as infection-induced cellular stress [38], cytoskeletal alterations, and membrane remodeling [39]. Additionally, *Rickettsia spp.* may inhibit apoptosis, allowing for continued bacterial replication and further contributing to the increase in cell size [40]. To obtain statistical significance for the thickness and diameters obtained by image analysis, statistical analysis was carried out using GraphPad Prism 10.2.3. as represented in Fig. 4. The diameter of the two cell populations was statistically different with a p-value of < 0.0001, whereas the membrane thickness was found to be non-significant.

Further, the TEM images demonstrate that the Vero cells have a vacuole and nucleus, but for the sake of simplicity and lack of other parameters in supporting research studies, we utilized the single-shell dielectric model to obtain the bioelectric signatures of these cells. It has been discussed elsewhere that mammalian cells can be accurately described by a single-shell dielectric model [41].

### Bioelectric signatures using single-shell dielectric model

Dielectric signatures were obtained using a 3DEP analyzer that applies non-uniform, radially axisymmetric electric fields to 20 well-shaped structures approximately 1 mm across and 2 mm deep. The cells were analyzed for up to 60 s at five points per decade (10 kHz–45 MHz) and all data were averaged before determining *σ*_*cyto*_ by fitting to the Clausius–Mossotti model. The averaged values were then fed into MATLAB to determine the properties listed in Table 1 using the single-shell model equations discussed earlier in the theory section (Sect. 2). The specific membrane capacitance (C_spmem_ = ε_mem_/d) and the specific membrane conductance (G_mem_ = σ_mem_/d ) are obtained from the 3DEP analyzer generated experimental data (Table 1) using the above equations, where d is the thickness of the shell (membrane), ε_mem,_ and σ_mem_ are the obtained membrane permittivity and membrane conductivity, respectively [42].

**Table 1:** Average dielectric properties and morphology of healthy and Rickettsia-infected Vero cells with *R* > 0.9 using a 3DEP analyzer at a suspending medium conductivity of 0.05 S/m. The healthy and infected cell populations have distinct bioelectric signatures that serve as biomarkers for designing an early detection tool for rickettsial infections

Parameter (unit)	Healthy Vero Cells	Rickettsia spp. Infected Cells
Cytoplasm Permittivity, ${\varepsilon _{cyto}}$ (F/m)	60	60
Cytoplasm Conductivity, σ_cyto_ (S/m)	0.14	0.08
Specific Membrane Capacitance, $\:\mathbf{C}$_spmem_ (F/m^2^)	0.0266	0.0113
Specific Membrane Conductance, G_mem_ (S/m^2^)	2025.57	3265.59
Whole Cell Capacitance (F)	3.69*10^− 12^	4.29*10^− 12^
Whole Cell Conductance (S)	0.28*10^− 6^	1.24*10^− 6^
Folding Factor, $\:\varphi\:$	2.9	1.7
First Crossover Frequency (kHz)	122.03	209
Second Crossover Frequency (MHz)	20	40
Diameter (µm)	6.65	11.11
Membrane Thickness (nm)	16	18

Another property of interest in this study is the membrane folding factor, $\:\varphi\:$, which describes the roughness or topography of a cell’s plasma membrane surface. The folding factor is expressed as the ratio of the actual membrane capacitance to that of a smooth membrane (ϕ = C_actual_/C_smooth_​), with the smooth membrane typically assumed to have a specific capacitance of 9.0 mF/m^2^. For intact cells, the DEP crossover frequency is dependent on the product of the cell radius *R*, the membrane folding factor ϕ, and the capacitance per unit area of the smooth membrane. This sensitive dependency of the dielectric phenotype on membrane folding, in addition to cell size, distinguishes DEP from other techniques used to sort cells that depend on size alone.

As demonstrated in Table 1, healthy Vero cells have a higher membrane folding factor, likely due to increased microvilli length, as suggested by previous studies. This increase in microvilli length results from the formation of short, branched filaments that mediate the development of long, parallel actin filaments characteristic of microvilli. In contrast, Rickettsia-infected cells exhibit decreased membrane folding, potentially driven by the changes including increased size or surface area of the infected cell and the increased number of microvilli along with the shortened length and the size (microvilli were plump) of the microvilli that adhered to the bacteria. Further, a ruffling membrane was also observed to cover the bacteria to be engulfed [43]. All these factors contributed to the decreased folding factor of infected cells compared to the intact healthy Vero cells.

**Fig. 5:**
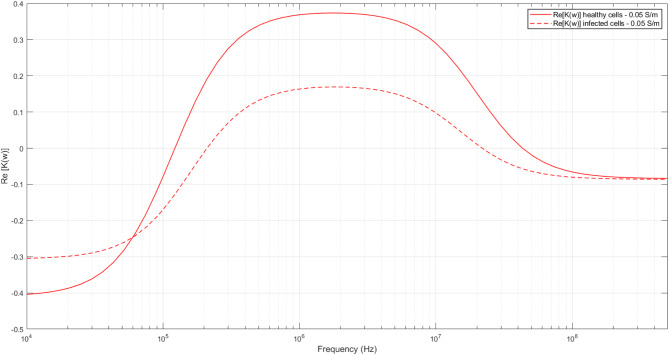
The plot of the real part of Clausius Mossotti (Re[K(w)]) for healthy and Rickettsia-infected Vero cells at 500 µS/cm suspending medium conductivity using the experimental data is shown. The solid line represents the plot for healthy Vero cells and the dotted line represents Rickettsia-infected Vero cells. At a fixed frequency, the healthy and infected cells experience different DEP forces that can be exploited for sorting or enriching the cells of interest

Additionally, experiments were conducted at various suspending medium conductivities, specifically 100, 200, 300, and 500 µS/cm, to understand the variations in bioelectric signatures between healthy and infected cells. MATLAB was utilized to input single-shell dielectric model equations to linearly fit the experimental data and further plot the real part of the Clausius-Mossotti (CM) factor at 500 µS/cm using properties determined in Table 1, as shown in Fig. 5. The crossover frequency, where the DEP force changes direction, is especially important as it plays a crucial role in enabling the separation of two cell populations [10, 11, 15, 44]. The suspending medium also influences the strength of the DEP force used for cell separation and should be selected based on the specific type of experiment [45]. It is evident from Fig. 5 that there are significant differences observed in the first and second crossover frequency between healthy and infected cells. The difference in the crossover frequency ranges, especially at ultra-high frequency regimes, can be utilized to design a rapid, sensitive, and label-free diagnostic platform that could detect infections at an early stage. The suspending medium conductivity with significant differences in the dielectric properties is chosen here, i.e., 0.05 S/m, and the dielectric spectra for other medium conductivities, i.e., 100, 200 and 300 µS/cm along with the calculated list of dielectric properties is provided in the supplementary document of the manuscript.

**Fig. 6:**
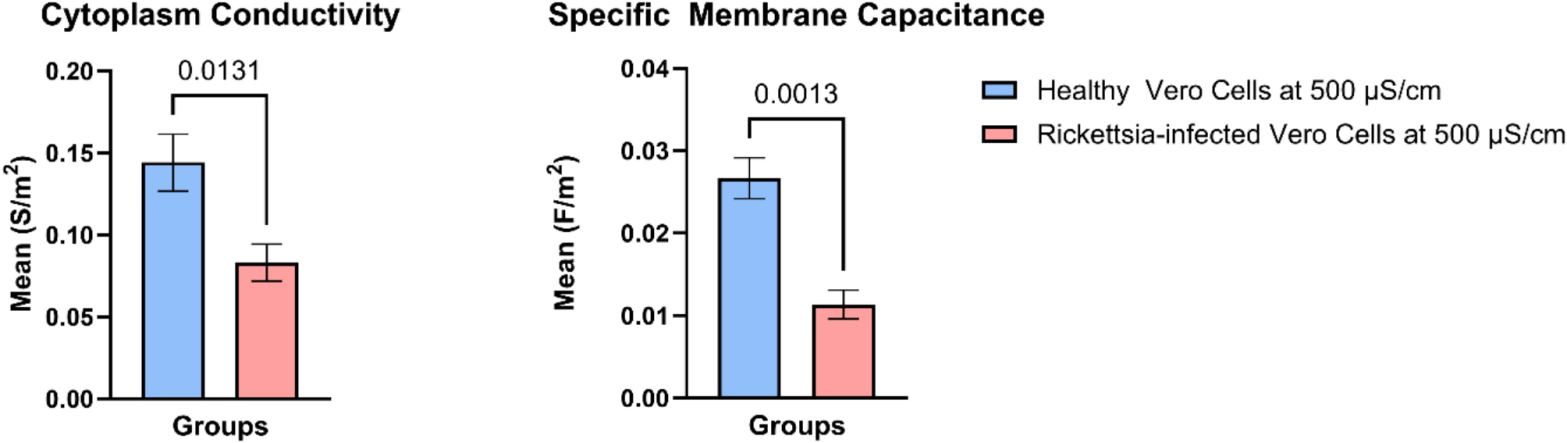
Error analysis and statistically significant differences in the cytoplasm conductivity and specific membrane capacitance for healthy Vero cells and Rickettsia-infected Vero cells at 500 µS/cm

Further, statistical analysis utilizing a paired t-test was conducted to obtain statistical significance of the fitted data. From Fig. 6 it is evident that the cytoplasm conductivity and the specific membrane capacitance of healthy and infected cells are significantly different at a suspending medium conductivity of 500 µS/cm. The corresponding p-values representing the statistical significance were manually added to the plot. Dielectric properties, such as conductivity (σ) and relative permittivity (ε), are intrinsic characteristics of biological cells and tissues and are crucial factors in the dissipation of electromagnetic (EM) energy within the human body [46].

Effective membrane capacitance represents the ability of the cell membrane to store electrical charge and is influenced by structural and morphological changes. Although the membrane’s composition typically remains constant, deviations from a smooth, spherical surface—such as blebs, microvilli, or invaginations—increase the effective surface area, thereby elevating membrane capacitance. This parameter serves as a sensitive indicator of cell surface morphology, with proportional increases in capacitance corresponding to increases in surface area.

Intracellular conductivity, on the other hand, reflects the ionic charge distribution within the cytoplasm and is influenced by factors such as ion channel activity, ion concentration, and membrane permeability. For instance, when the cell membrane becomes permeable, the formation of pores establishes a connection between the intracellular and extracellular environments, resulting in a rapid decrease in intracellular conductivity. This property is a critical biomarker for assessing cellular responses, such as apoptosis, where ion efflux and water loss markedly alter cytoplasmic conductivity.

Rickettsial invasion of host cells is a dynamic process involving a complex interplay between the pathogen and host cellular mechanisms. Intracellular bacteria actively modulate membrane dynamics, actin cytoskeleton remodeling, phosphoinositide (PI) metabolism, and intracellular trafficking to facilitate entry, survival, and proliferation within the host [47]. These interactions not only disrupt the structural integrity of the host cell membrane and cytoskeleton but also induce physiological changes, including alterations in ion flux and intracellular signaling pathways. Collectively, these structural and physiological modifications can have a profound impact on the dielectric properties of infected cells.

The dielectric properties of healthy and infected cells, as shown in Table 1; Fig. 6, differ significantly at 500 µS/cm, causing them to experience different DEP forces at certain frequencies. The data shows that Rickettsia-infected Vero cells demonstrate a unique bioelectrical signature that can be distinguished from their healthy intact counterpart Vero cells at 99% confidence.

## Conclusions

Spotted Fever, caused by *Rickettsia spp.*, is a life-threatening bacterial disease in humans when left untreated. It is crucial to detect this disease early to begin treatment for the best prognosis. The gold standard for diagnosing spotted fever is indirect immunofluorescent assay (IFA) or polymerase chain reaction assay (PCR). These techniques are costly and can take up to two weeks to accurately diagnose them. To overcome these disadvantages, we utilize dielectrophoresis which offers many advantages including label-free whole-cell characterization of cells preserving their viability. DEP also enables real-time analysis and manipulation of cells in their native state, allowing observation of dynamic cellular responses to non-uniform electric fields.

Here, to identify bioelectric signatures (biomarkers), we characterize and analyze cell behavior utilizing the DEP crossover technique and a single-shell dielectric model to fit the experimental data generated using a 3DEP analyzer. This study utilizes healthy Vero cells and those inoculated with *Rickettsia montanensis* to obtain a quicker, more cost-efficient, and sensitive detection method for this bacterial disease. The differences in cellular parameters of healthy versus infected cells were characterized, particularly the cytoplasm conductivity, and specific membrane capacitance. A significant difference was demonstrated at a medium conductivity of 500 µS/cm as shown in Fig. 6. Inoculated Vero cell morphology varies from that of healthy Vero cells due to the infection of *Rickettsia spp.* The differences in the bioelectrical signatures observed show the correlation with the membrane morphology and cytoplasmic content of these infected cells.

The differences in the dielectric (Clausius-Mossotti factor) spectrum were found to be unique and statistically significant at a medium conductivity of 500 µS/cm and a p-value of < 0.01. This plot yields the first and second crossover frequencies of both healthy and infected Vero cells, which were shown to be significantly different. The data at this medium conductivity (0.05 S/m) would be ideal to use for further sorting of the two cell populations as the crossover frequencies are uniquely distinct. This data indicates that *Rickettsia spp.* infection could be detected electrically without the need for PCR assay in a quick, cost-effective, sensitive way.

These dielectric signatures could potentially be utilized to design a microfluidic, lab-on-a-chip diagnostic tool that could non-invasively detect rickettsial infections early and quickly in a label-free way. To improve the diagnostic tool’s sensitivity and specificity, various strains of *Rickettsia* spp. and different levels of infection (early, mid, and late) will be analyzed to capture distinct bioelectric signatures, enabling a broader detection spectrum for the disease. Given the potential variation in biomarkers among different *Rickettsia* strains, optimizing the device to accurately detect a wide range of strains is critical for comprehensive and reliable diagnostics. Further, to enhance the sensitivity and efficiency of detection, the two-shell dielectric model will be utilized to fit the experimental data rather than the single-shell dielectric model. This innovative approach holds significant promise for transforming the diagnosis of spotted fever and related bacterial infections, offering a faster, more accessible solution, especially in low-resource settings for early disease detection.

## Electronic supplementary material

Below is the link to the electronic supplementary material.

Supplementary Material 1

## Data Availability

The datasets generated during and/or analyzed during the current study are available from the corresponding author upon reasonable request.
