# Supplementary material for: Bioelectric profiling of Rickettsia montanensis in Vero cells utilizing dielectrophoresis
Source: J Biol Eng. 2025 Feb 18;19:18. doi: 10.1186/s13036-025-00487-y (PMC11837300; doi:10.1186/s13036-025-00487-y)
Supplement: Supplementary file 1 — Supplementary Material 1 [file 13036_2025_487_MOESM1_ESM.docx]

**Supplementary Information**

Table 1. List of dielectric properties of healthy and infected Vero cells at 100, 200 and 300 µS/m.

| Parameter (unit) | Healthy | Infected | Healthy | Infected | Healthy | Infected |
| --- | --- | --- | --- | --- | --- | --- |
| Conductivity (S/m) | 0.01 S/m | | 0.02 S/m | | 0.03 S/m | |
| Cytoplasm permittivity, $\boldsymbol{\varepsilon}$_cyto_ (F/m) | 60 | 60 | 60 | 60 | 60 | 60 |
| Cytoplasm conductivity, σ_cyto_ (S/m) | 0.29 | 0.16 | 0.105 | 0.04 | 0.17 | 0.22 |
| Specific membrane capacitance, $\mathbf{C}$_spmem_ (F/m^2^) | 0.016 | 0.016 | 0.019 | 0.0103 | 0.026 | 0.016 |
| Specific membrane conductance, G_mem_ (S/m^2^) | 1347.69 | 624.02 | 975.23 | 1405.94 | 2778.48 | 2087.11 |
| Whole cell capacitance, (F) | 2.2e-12 | 6.08e-12 | 2.6e-12 | 3.91e-12 | 3.61e-12 | 6.08e-12 |
| Whole cell conductance, (S) | 0.18e-6 | 0.23e-6 | 0.13e-6 | 0.5e-6 | 0.3e-6 | 0.7e-6 |
| Folding factor, $\boldsymbol{\phi}$ | 1.7 | 1.7 | 2.1 | 1.1 | 2.8 | 1.7 |
| Crossover frequency (KHz) | 34.8 | 21.84 | 65.2 | 82.2 | 68.59 | 64.6 |


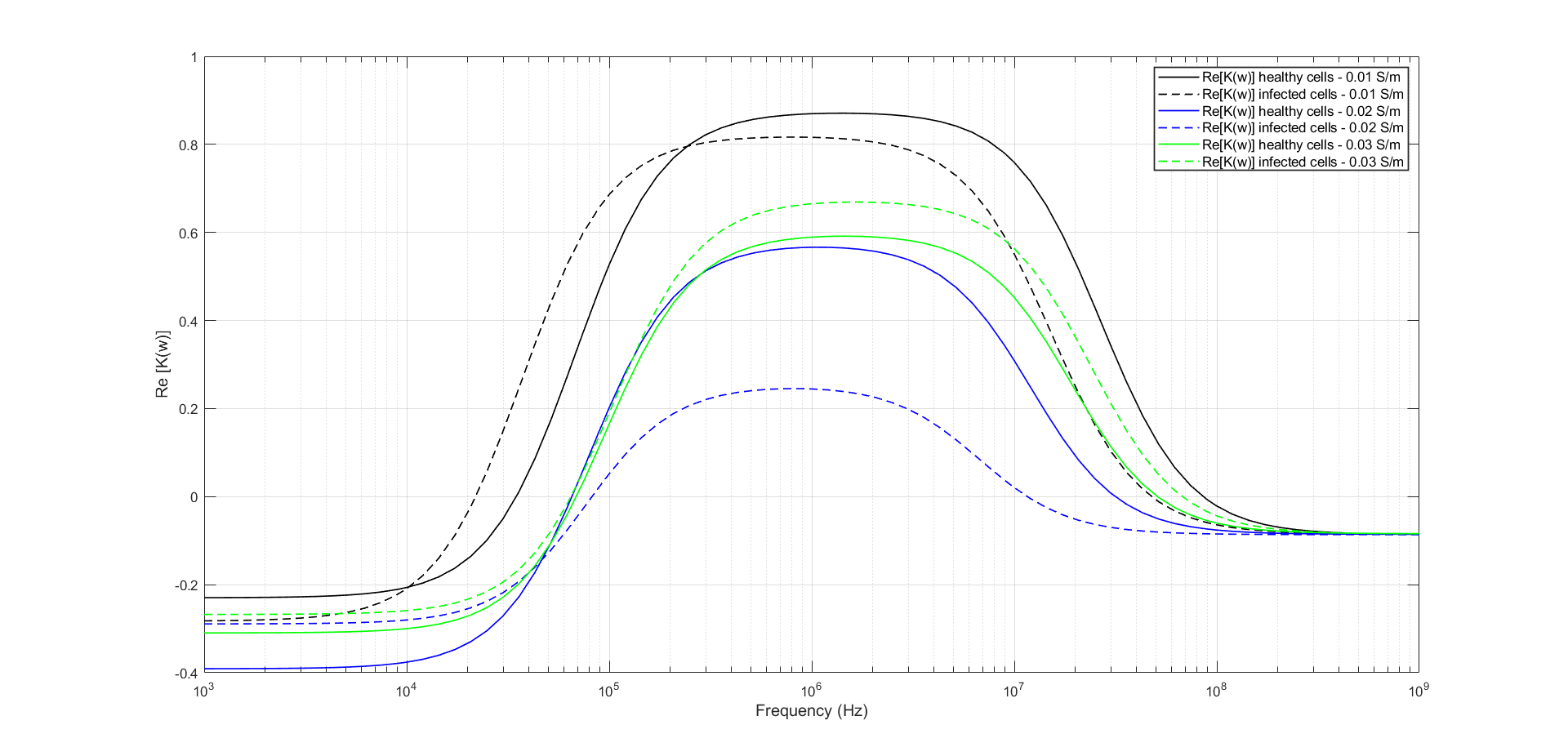


Figure 1. Plot for Clausius Mossotti factor of healthy and infected Vero cells at 100, 200 and 300 µS/m.


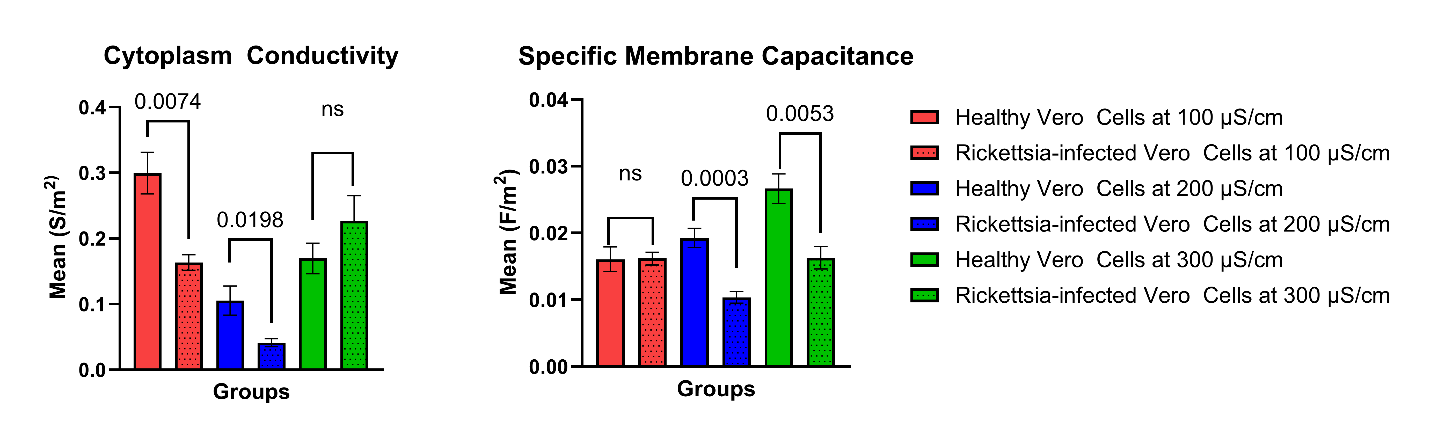


Figure 2. Statistical analysis for cytoplasm conductivity and specific membrane capacitance of healthy and infected Vero cells at 100, 200 and 300 µS/m.
